# Effects of Acceptance and Commitment Therapy on Self‐Compassion, Self‐Criticism, and Emotional Well‐Being in Adults With Mental Health Concerns: A Systematic Review

**DOI:** 10.1002/hsr2.72525

**Published:** 2026-05-25

**Authors:** Andrea Calderone, Desirèe Latella, Giulia Marafioti, Caterina Formica, Angela Foti, Elvira la Fauci, Giuseppa Filippello

**Affiliations:** ^1^ University of Messina Messina Italy; ^2^ I.R.C.C.S Centro Neurolesi Bonino Pulejo Messina Italy

**Keywords:** acceptance and commitment therapy, mental health, psychological flexibility, self‐compassion, self‐criticism

## Abstract

**Background and Aims:**

Self‐compassion is a supportive self‐relating style linked to emotional well‐being. Acceptance and Commitment Therapy (ACT) targets psychological flexibility and may influence self‐compassion, yet evidence is inconsistently quantified. This systematic review investigated changes in self‐compassion following ACT or ACT‐based interventions in adults with mental health concerns and summarized findings for self‐criticism and emotional well‐being.

**Methods:**

We followed PRISMA 2020 and preregistered the protocol on the Open Science Framework (10.17605/OSF.IO/8u7e9). PubMed, Embase, Web of Science, and the Cochrane Library were searched to August 5, 2024. Two reviewers (A.C., D.L.) independently screened studies and extracted data. C.F. adjudicated discrepancies, with substantial agreement at title and abstract screening (*κ* = 0.72) and full‐text eligibility (*κ* = 0.78). RoB 2 and ROBINS‐I were used for randomized and non‐randomized studies. We synthesized results narratively following SWiM and reported effect estimates with uncertainty when available. Meta‐analysis was not performed.

**Results:**

Ten studies were included (five randomized controlled trials and five non‐randomized, experimental, or qualitative studies). Self‐compassion was most often measured with the self‐compassion scale. In trials, within‐group improvements were common, but between‐group effects were mixed, ranging from negligible contrasts to large gains in selected samples (one trial reported a large within‐group change, *d* ≈ 1.5, versus a smaller change in the waitlist). A pre–post chronic pain evaluation reported a small improvement in self‐compassion (*d* ≈ 0.21). Self‐criticism was reported in one trial with minimal post‐treatment separation, and emotional well‐being outcomes were variably defined and mixed. Most randomized controlled trials had some concerns of bias (one high risk), and most non‐randomized studies were at serious risk or provided insufficient information.

**Conclusion:**

ACT may improve self‐compassion, but comparative effects are inconsistent and certainty is limited. Standardized measurement and complete reporting are needed to clarify durability and clinical relevance.

## 1 Introduction

Mental health disorders and clinically significant psychological distress contribute to disability and impaired functioning worldwide [1]. Large surveys indicate that mental disorders are common in adulthood and can disrupt education, work participation, and social functioning [2]. Recent crises have reinforced the need for scalable interventions that address transdiagnostic processes linked to symptom persistence [3]. Chronic stress is one such process and can increase vulnerability to depression and anxiety [4, 5]. Psychological flexibility, defined as the capacity to remain in contact with present‐moment experience while pursuing valued behavior, has been proposed as a modifiable process relevant across disorders [6]. Acceptance and Commitment Therapy (ACT) is a process‐based behavioral intervention grounded in the psychological flexibility model and organized around six interrelated processes often described in the ACT hexaflex [7]. Evidence syntheses support ACT across several mental and physical health problems, although effects vary and heterogeneity is expected [8, 9]. Psychological flexibility is commonly assessed with validated measures such as the Acceptance and Action Questionnaire‐II [10].

Self‐compassion has emerged as a clinically meaningful construct that may shape how individuals respond to distress and perceived inadequacy. Self‐compassion reflects a caring and nonjudgmental stance toward oneself during suffering, supported by mindful awareness and an understanding that imperfection is part of shared human experience [11]. This construct is distinct from generic “sensitivity” or interpersonal empathy because it is self‐directed and can be operationalized with validated instruments. Self‐compassion is frequently measured using the self‐compassion scale and its validated short form [12]. Cross‐cultural psychometric studies support the use of these measures in diverse settings [13]. Meta‐analytic evidence indicates that higher self‐compassion is associated with greater well‐being and lower psychological distress [14].

Self‐compassion is often considered alongside self‐criticism and emotional well‐being because these constructs capture different facets of self‐relating and positive mental health. Self‐criticism describes harsh self‐evaluation and punitive self‐relating that can amplify distress and interfere with coping [15]. Measurement work suggests that self‐compassionate responding relates to mood repair and emotion regulation, whereas self‐criticism can maintain negative affect [16]. Evidence indicates that self‐compassion and mindfulness may relate to anxiety and depression partly through resilience pathways [17]. Associations with perceived stress and health‐promoting behavior provide an additional rationale for clinical groups with comorbid physical conditions [18]. Findings in younger samples also show associations between self‐compassion and lower anxiety and depressive symptoms, although measures differ and construct clarity is essential for synthesis [19]. Recent studies suggest that self‐compassion can sit on pathways linking stress and anxiety to depression and that well‐being indicators may capture related outcomes [20, 21]. Work examining shame and guilt as mediators points to plausible affective mechanisms relevant to anxiety and depression presentations [22]. Emotional well‐being outcomes are also salient in adolescents and young adults, where self‐compassion has been linked to broader indicators of adjustment [23].

**Figure 1:**
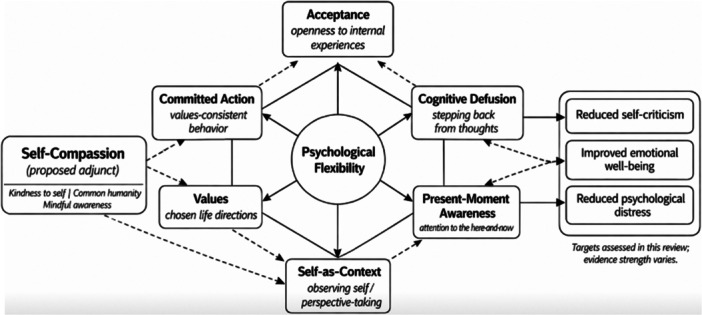
Proposed placement of self‐compassion within the ACT hexaflex model. The central node represents psychological flexibility. The six surrounding nodes represent core ACT processes: acceptance, cognitive defusion, present‐moment awareness, self‐as‐context, values, and committed action. Solid arrows indicate ACT model relationships. The self‐compassion node (kindness to self, common humanity, mindful awareness) is shown as a proposed adjunct process; dashed arrows indicate theory‐informed links to selected ACT processes and to review outcomes. Outcomes (reduced self‐criticism, improved emotional well‐being, reduced psychological distress) reflect targets assessed in this review; evidence strength varies across included studies. Abbreviation: ACT, acceptance and commitment therapy.

Taken together, this evidence indicates that self‐compassion, self‐criticism, psychological flexibility, and emotional well‐being are conceptually related but not interchangeable clinical constructs. ACT is relevant to this evidence base because its core processes may influence how individuals relate to distressing self‐evaluations. Acceptance and present‐moment awareness may reduce experiential avoidance and support balanced awareness of distress. Cognitive defusion and self‐as‐context may reduce fusion with self‐critical content. Values clarification and committed action may support behavior aligned with self‐care and meaningful living. Empirical work links psychological flexibility, self‐compassion, and emotional well‐being, and randomized and applied studies suggest that ACT or ACT‐informed interventions can increase self‐compassion in some contexts [24, 25, 26, 27, 28, 29, 30]. However, no systematic review has specifically synthesized whether ACT or ACT‐based interventions improve self‐compassion in adults with mental health concerns or how self‐criticism and emotional well‐being have been operationalized in this literature. This systematic review aimed to synthesize evidence on changes in self‐compassion following ACT or ACT‐based interventions in adults with mental health concerns. Secondary aims were to summarize changes in self‐criticism and emotional well‐being when reported and to describe how these constructs were operationalized across studies. To support interpretation, we propose a theory‐informed integration of self‐compassion within the ACT psychological flexibility model (hexaflex), highlighting plausible links to core processes and the outcomes assessed in this review (Figure 1).

## 2 Materials and Methods

### 2.1 Review Design, Protocol Record, and Reporting Approach

2.1

We conducted a systematic review to synthesize evidence on changes in self‐compassion following Acceptance and Commitment Therapy (ACT) or ACT‐based interventions in adults with mental health concerns. The review question and eligibility criteria were framed using the PICO approach [31]. Reporting followed the Preferred Reporting Items for Systematic Reviews and Meta‐Analyses (PRISMA) 2020 statement, and we used PRISMA guidance to support transparent description of the search, screening, and synthesis workflow [32, 33]. When statistical pooling was not appropriate, we structured the narrative synthesis using the Synthesis Without Meta‐analysis (SWiM) reporting guideline [34]. The review was preregistered on the Open Science Framework (OSF; DOI: 10.17605/OSF.IO/8u7e9) on July 31, 2024. Following peer‐review revisions, we uploaded and updated the full protocol document on February 11, 2026.

### 2.2 Eligibility Criteria

Eligible studies enrolled adults aged 18 years or older with mental health concerns. We defined mental health concerns as a diagnosed mental disorder or clinically significant psychological distress as described by the study authors. We also considered studies conducted in adults with chronic medical conditions or caregiving roles when psychological outcomes were a primary focus, and participants experienced clinically relevant distress. The intervention of interest was ACT or ACT‐based intervention programs delivered in any format, including individual, group, or digitally delivered interventions. Interventions were considered ACT‐based when authors explicitly described the program as grounded in ACT and the psychological flexibility model and when the content targeted core ACT processes in a structured manner. Comparators were not restricted and could include treatment as usual, waitlist, active control conditions, other psychotherapies, or within‐participant pre‐post comparisons.

The primary outcome was self‐compassion measured with a validated instrument. Secondary outcomes included self‐criticism and emotional well‐being when assessed with validated measures and reported as outcomes of the ACT intervention. Studies were eligible if they reported at least one of these outcomes at baseline and at one or more post‐intervention time points. Symptom outcomes such as depression, anxiety, and stress were extracted when reported alongside the outcomes of interest to support clinical interpretation, but symptom outcomes were not required for inclusion.

We included randomized controlled trials and non‐randomized intervention studies that evaluated ACT or ACT‐based interventions and reported outcome data. Single‐arm pre‐post studies were eligible when they evaluated an ACT‐based intervention and provided quantitative outcome data. We prespecified two complementary evidence streams. The intervention evidence stream included randomized and non‐randomized studies evaluating ACT or ACT‐based interventions and reporting self‐compassion outcomes. The contextual/process evidence stream included observational, experimental, or qualitative studies that examined associations between self‐compassion and ACT‐relevant constructs (e.g., psychological flexibility/inflexibility, experiential avoidance, cognitive fusion, or perspective‐taking), without requiring delivery of an ACT intervention. Findings from contextual/process studies were synthesized descriptively and were not interpreted as estimates of intervention efficacy. We excluded protocols without outcome data, conference abstracts without full text, dissertations, and narrative, integrated, or systematic reviews. Inclusion was limited to human studies published in English with available full text.

### 2.3 Information Sources and Search Strategy

We searched PubMed, Embase, Web of Science, and the Cochrane Library from inception to August 5, 2024. The core search strategy combined (All Fields: “Acceptance and Commitment Therapy”) AND (All Fields: “Self‐Compassion”), and the syntax was adapted to each database interface. No restrictions were applied to publication year. We also screened the reference lists of included studies and relevant reviews to identify additional eligible records.

### 2.4 Study Selection and Data Management

Search results were collated and managed in Microsoft Excel. Duplicate records were removed prior to screening using title, author, and digital object identifier fields when available. Two reviewers (A.C. and D.L.) independently screened titles and abstracts against the eligibility criteria. Full texts were retrieved for records judged potentially eligible by either reviewer and were assessed independently by the same reviewers. Discrepancies were resolved through discussion, and a third reviewer (C.F.) adjudicated unresolved cases, including final decisions on records that were observational, process‐focused, or otherwise ambiguous with respect to the intervention requirement. Interrater agreement was quantified using Cohen's kappa, with substantial agreement at title and abstract screening (*κ* = 0.72) and at full‐text assessment (*κ* = 0.78) [35]. Study selection decisions were documented using a PRISMA 2020 flow diagram. Reasons for full‐text exclusion were recorded in the screening file to support transparency and to enable reproducibility of eligibility decisions.

### 2.5 Data Extraction and Data Items

Data extraction was performed in Microsoft Excel using a standardized form developed and piloted for this review. Two reviewers (A.C. and D.L.) extracted data independently and compared entries to ensure consistency, with consensus discussion and third‐reviewer adjudication (C.F.) when needed. Extracted items included study design; country and setting; participant characteristics, eligibility criteria, and sample size; mental health concern definition; intervention format, duration, and delivery characteristics; comparator details; follow‐up duration; and outcome measures. We also extracted information on intervention fidelity, adherence, therapist training, or supervision when reported, attrition, and adverse events or unintended effects when these were described.

For outcomes, we extracted the instrument used to measure self‐compassion, self‐criticism, and emotional well‐being; time points; and the group‐level data required to quantify change. ACT‐consistent process measures, including psychological flexibility or inflexibility, cognitive fusion, perspective‐taking, resilience, and related distress or symptom outcomes, were extracted only when reported alongside the review outcomes and were used as contextual information to interpret possible mechanisms rather than as additional efficacy endpoints. For controlled studies, we prioritized between‐group effects at the first post‐intervention assessment. For single‐arm designs, we summarized within‐participant change from baseline to post‐intervention. When follow‐up assessments were reported, we extracted follow‐up estimates as additional time points. When multiple intervention arms were eligible, we extracted data for each eligible arm and ensured that comparisons did not double‐count participants in any pooled synthesis.

We extracted effect estimates and measures of uncertainty when reported, including mean differences, standardized mean differences, odds ratios, or risk ratios with corresponding confidence intervals. When sufficient information was provided, we derived effect estimates from descriptive statistics. When key outcome data required for synthesis were unclear or missing, we attempted to contact study authors when feasible.

### 2.6 Risk of Bias Assessment

Risk of bias was assessed independently by two reviewers (A.C. and D.L.) at the study level. Randomized controlled trials were evaluated using the revised Cochrane risk of bias tool for randomized trials (RoB 2) [36]. Non‐randomized studies of interventions were evaluated using the ROBINS‐I tool [37]. Discrepancies were resolved through discussion and, when necessary, adjudication by a third reviewer (C.F.). Risk of bias judgments were used to support interpretation, particularly when effect estimates were imprecise or when confounding or selective reporting was likely.

### 2.7 Data Synthesis and Presentation

We anticipated clinical and methodological heterogeneity across populations, intervention formats, and measurement tools. We therefore conducted a primarily narrative synthesis structured by population context, intervention format, and outcomes. Synthesis methods were reported following SWiM guidance, including explicit grouping decisions, transparent presentation of study‐level findings, and a structured approach to describing the direction and consistency of effects [34]. We did not perform statistical pooling or additional quantitative analyses. When studies reported effect estimates and uncertainty (e.g., standardized mean differences or mean differences with confidence intervals), these were presented as reported. When such metrics were not available, we summarized outcome changes qualitatively while maintaining clear distinctions between controlled comparisons and pre‐post within‐group findings.

We explored the influence of study design and risk of bias through structured grouping and narrative comparison rather than formal sensitivity analyses. When studies reported multiple post‐intervention time points, we treated the first post‐intervention assessment as the primary time point for synthesis and described follow‐up findings separately when available.

## 3 Results

### 3.1 Study Selection

**Figure 2:**
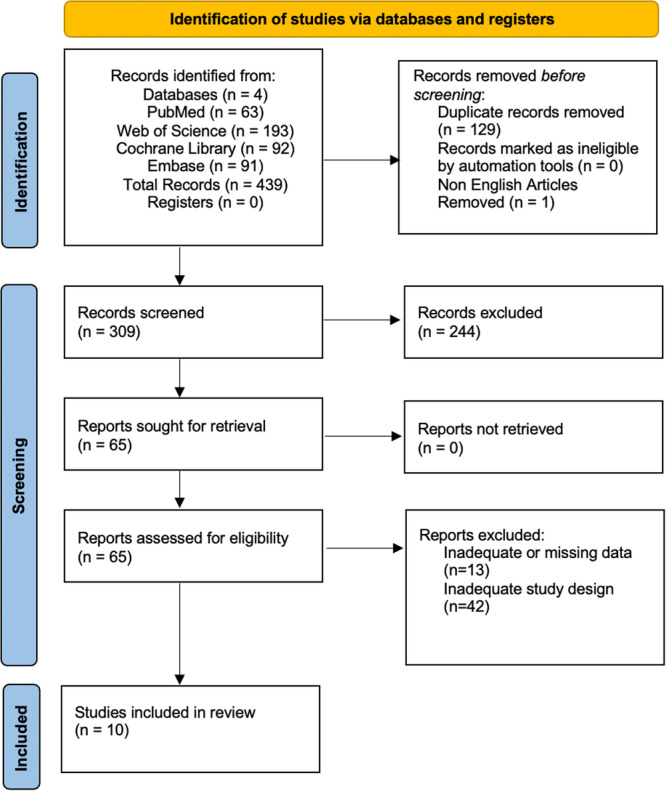
PRISMA 2020 flow diagram. Records were identified from PubMed (*n* = 63), Web of Science (*n* = 193), Cochrane Library (*n* = 92), and Embase (*n* = 91), for a total of 439 records. Before screening, duplicates were removed (*n* = 129) and one non‐English record was excluded (*n* = 1), leaving 309 records for title and abstract screening; 244 records were excluded. Full texts were assessed for 65 reports; none were not retrieved. Fifty‐five reports were excluded due to inadequate or missing data (*n* = 13) or inadequate study design (*n* = 42). Ten studies were included in the review.

Database searches identified 439 records (PubMed *n *= 63, Web of Science *n *= 193, Cochrane Library *n *= 92, Embase *n *= 91). Before screening, 129 duplicate records were removed, and 1 non‐English record was excluded, leaving 309 records for title and abstract screening. We excluded 244 records at this stage and sought the retrieval of 65 full‐text reports; all reports were retrieved. After full‐text assessment, 55 reports were excluded due to inadequate or missing data (*n *= 13) or inadequate study design (*n *= 42), resulting in 10 studies included in the review. The study selection process is summarized in Figure 2.

### 3.2 Risk of Bias

**Figure 3:**
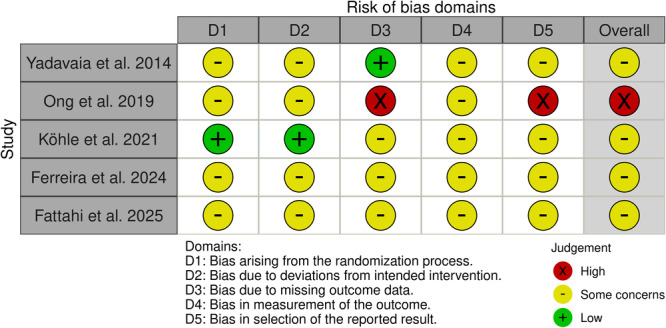
Risk of bias domains for randomized controlled trials (RoB 2). Traffic‐light plot summarizing domain‐level and overall RoB 2 judgments for included randomized controlled trials. Domains are: D1, bias arising from the randomization process; D2, bias due to deviations from intended interventions; D3, bias due to missing outcome data; D4, bias in measurement of the outcome; D5, bias in selection of the reported result. Symbols indicate judgments: green (+), low risk of bias; yellow (–), some concerns; red (X), high risk of bias. “Overall” reflects the RoB 2 overall judgment for each study. Abbreviations: RCT, randomized controlled trial; RoB 2, Risk of Bias 2.

Five studies were randomized controlled trials (RCTs) and were assessed with the revised Cochrane Risk of Bias tool (RoB 2). Four RCTs were judged as having some concerns of bias overall (Yadavaia et al. [25], Köhle et al. [38], Ferreira et al. [29], and Fattahi et al. [30]), and one RCT was judged as having a high risk of bias overall (Ong et al. [39]). Across the RCTs, the most common concerns related to potential deviations from intended interventions, missing outcome data, and outcome measurement and reporting (see Figure 3).

**Figure 4:**
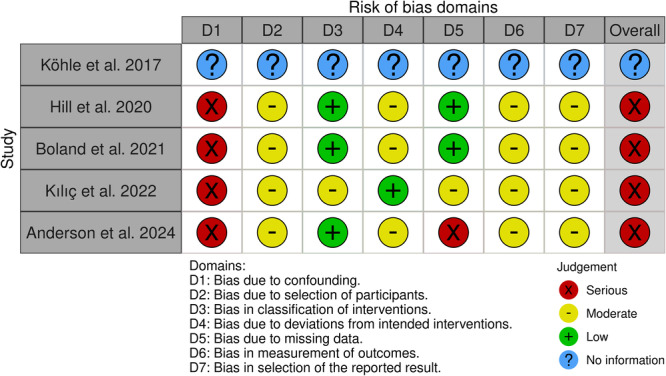
Risk of bias domains for non‐randomized studies (ROBINS‐I). Traffic‐light plot summarizing domain‐level and overall ROBINS‐I judgments for included non‐randomized studies. Domains are: D1, bias due to confounding; D2, bias due to selection of participants; D3, bias in classification of interventions; D4, bias due to deviations from intended interventions; D5, bias due to missing data; D6, bias in measurement of outcomes; D7, bias in selection of the reported result. Symbols indicate judgments: green (+), low risk; yellow (–), moderate risk; red (X), serious risk; blue (?), no information. “Overall” reflects the ROBINS‐I overall judgment for each study. Abbreviation: ROBINS‐I, risk of bias in non‐randomized studies of interventions.

Five studies were nonrandomized (including qualitative and experimental designs) and were assessed with the Risk Of Bias In Non‐randomized Studies of Interventions (ROBINS‐I). Four nonrandomized studies were judged as having a serious overall risk of bias (Hill et al. [40], Boland et al. [41], Anderson et al. [26], and Kılıç et al. [27]) largely due to confounding and selection bias, and one qualitative study provided insufficient information to determine risk of bias (Köhle et al. [28]). Overall, the body of evidence was limited by heterogeneity of study designs and outcomes and by risk of bias that reduced confidence in causal inference, particularly for nonrandomized findings (see Figure 4).

### 3.3 Study Characteristics

Of the 10 included studies, five evaluated ACT or ACT‐based interventions and five provided contextual/process evidence on links between self‐compassion and ACT‐relevant constructs. Study characteristics are summarized in Table 1 and an overview of validated instruments used to assess self‐compassion, compassion, and closely related constructs is summarized in Table S1. The 10 included studies were heterogeneous in population, intervention format, and outcome reporting. The five RCTs targeted distressed undergraduates selected for low self‐compassion (a brief ACT workshop) [25], adults with clinical perfectionism (a 10‐session ACT intervention) [39], partners of patients with cancer (a web‐based ACT self‐help intervention delivered with either personal feedback or automated feedback) [38], adults with inflammatory bowel disease (ACT group intervention; follow‐up to 12 months) [29], and adults with cardiovascular disease (ACT group intervention; follow‐up to 2 months) [30]. The nonrandomized evidence consisted of one qualitative study in partners of cancer patients [28], one multiple‐baseline single‐case study in women with restrictive eating and body checking [40], one large pre‐post evaluation of an ACT‐based chronic pain program [26], one laboratory experiment on perspective‐taking exercises aligned with ACT‐consistent processes [41], and one longitudinal observational study investigating psychological inflexibility, self‐compassion, and distress among adults with type 2 diabetes [27].

**Table 1:** Summary of included studies.

Author et al., year, location/country	Aim	Study design/intervention/treatment period	Sample size/sample characteristics	Outcome measures	Main findings	Effect size (or *p* value)/certainty of evidence	Safety and adverse events	ACT intervention/delivery mode (and comparator)	Self‐compassion and key process outcomes (measures and main change)
Author: Anderson et al. [26] Year: 2024 Location/country: London, United Kingdom	To examine changes in stigma and self‐compassion during ACT‐based residential pain management and their associations with pain‐related outcomes.	Design: pre–post cohort (observational) Arms: single group (no comparator) Dose and period: residential pain management treatment based on ACT; 3 weeks (4 days/week) Timepoints: pre‐ and post‐treatment	*N* randomized/analyzed: NR (non‐randomized); *N *= 519 baseline; *N *= 431 post‐treatment Population: adults with chronic pain (pain duration mean 13.61 ± 11.21 years) Age/mean: 48.18 ± 13.04 years (missing = 106) Sex (M/F): 109/407 (missing = 3)	Clinical outcomes: pain intensity; pain interference (BPI‐IS); work and social adjustment (WSAS); depression (PHQ‐9) Process outcomes: self‐compassion (SCS‐SF); stigma (SSCI‐8); pain acceptance (CPAQ‐8); cognitive fusion (CFQ‐7); committed action (CAQ‐8); self‐as‐context (SEQ)	Self‐compassion and most pain outcomes improved from pre‐ to post‐treatment. Stigma, committed action, and cognitive fusion did not significantly change.	Effect size (or *p* value): self‐compassion: *d *=0.21, *p *< 0.001; pain intensity: *d* = 0.49, *p*< 0.001; pain interference: *d *= 0.89, *p *< 0.001; depression: *d *= 0.88, *p *< 0.001; stigma: *p *= 0.128 (*d *= 0.08) Certainty of evidence/RoB: ROBINS‐I (overall: serious)	Adverse events: NR Dropouts: Missing post‐treatment data: 88/519 (17.0%)	ACT intervention: interdisciplinary ACT‐based residential pain management program Delivery mode: in‐person, residential (3 weeks; 4 days/week) Comparator: none (pre–post)	Self‐compassion: SCS‐SF; small increase (*d *= 0.21, *p *< 0.001) Key ACT processes: Acceptance (CPAQ‐8) ↑ (*d* = 0.42, *p*< 0.001); self‐as‐context (SEQ) ↑ (*d *= 0.21, *p *< 0.001); committed action (CAQ‐8) NR/no sig. change; cognitive fusion (CFQ‐7) NR/no sig. change
Author: Boland et al. [41] Year: 2021 Location/country: United Kingdom	To test whether brief deictic (perspective‐taking) framing exercises affect emotional discomfort, cognitive fusion, and self‐compassion.	Design: experimental mixed design (single‐session) Arms: between: interpersonal vs. temporal deictic framing; Within: baseline/control/giving compassion/receiving compassion Dose and period: single lab session Timepoints: repeated measures within session	*N* randomized/analyzed: NR; *N *= 61 Population: community/university sample Age/mean: 27.87 years (SD = 0.82), range 22–60 (as reported) Sex (M/F): 13/48	Outcomes: emotional discomfort (0–100); state cognitive fusion scale; state self‐compassion Scale	Across conditions, giving/receiving compassion was associated with reduced discomfort and cognitive fusion and increased state self‐compassion. Some interaction effects by deictic framing type were observed.	Effect size (or *p* value): state self‐compassion: *F*(3,177) = 54.19, *p *< 0.001, *η*² = 0.48; cognitive fusion: *F*(3,177) = 45.11, *p *< 0.001, *η*² = 0.43; discomfort: *F*(3,177) = 51.16, *p *< 0.001, *η*² = 0.46 Certainty of evidence/RoB: ROBINS‐I (overall: serious)	Adverse events: NR Dropouts: NR	ACT intervention: No formal ACT; brief deictic/perspective‐taking exercise grounded in ACT/RFT concepts Delivery mode: in‐person experimental task (single session) Comparator: Control/baseline condition; interpersonal vs. temporal framing	Self‐compassion: state self‐compassion scale; increased within session (*F* = 54.19, *p *< 0.001) Key processes: state cognitive fusion ↓ (*F* = 45.11, *p *< 0.001); emotional discomfort ↓ (*F *= 51.16, *p *< 0.001)
Author: Fattahi et al. [30] Year: 2025 Location/country: Iran	To evaluate the effect of group ACT on depression, anxiety, stress, emotion regulation, and self‐compassion in patients with cardiovascular disease.	Design: randomized controlled trial (parallel groups) Arms: ACT vs. control Dose and period: 8 group sessions; 90 min each Timepoints: pre, post, 2‐month follow‐up, 4‐month follow‐up	*N* randomized/analyzed: Randomized *N* = 40 (20 ACT; 20 control); post‐test: 18/19; follow‐up: 18/18 Population: adults with cardiovascular disease Age/mean: ACT 45.85 ± 5.39; control 46.40 ± 6.85 Sex (M/F): ACT 12/8; control 13/7	Clinical outcomes: depression, anxiety, stress (DASS‐21) Process outcomes: emotion regulation (ERQ: reappraisal, suppression); self‐compassion (SCS)	Compared with control, ACT showed significant improvements in depression, anxiety, stress, emotion regulation, and self‐compassion, maintained at follow‐ups.	Effect size (or *p* value): between‐group mean differences (post): depression −2.95 (*p *= 0.01); anxiety −2.50 (*p *= 0.01); stress −4.77 (*p *= 0.01); reappraisal +2.90 (*p *= 0.01); suppression −3.02 (*p *= 0.01); self‐compassion +5.16 (*p *= 0.01). Between‐subjects partial *η*² (group): self‐compassion 0.65 Certainty of evidence/RoB: RoB 2 (overall: some concerns)	Adverse events: NR Dropouts: post‐test *n *= 19 control, *n *= 18 ACT; follow‐up *n *= 18 in each arm	ACT intervention: group ACT (protocol based on Eifert and Forsyth, as reported) Delivery mode: in‐person group sessions Comparator: control group (NR: type of control intervention)	Self‐compassion: SCS; increased vs. control (*p *= 0.01) Key processes: emotion regulation: reappraisal ↑, suppression ↓ (both *p *= 0.01)
Author: Ferreira et al. [29] Year: 2024 Location/country: Portugal	To assess acceptability and preliminary efficacy of LIFEwithIBD, a face‐to‐face group ACT + compassion‐based intervention, for adults with inflammatory bowel disease.	Design: randomized controlled trial (parallel groups) Arms: LIFEwithIBD (ACT + compassion) vs. treatment as usual/control Dose and period: face‐to‐face group intervention over 9 weeks (number of sessions NR) Timepoints: T0 baseline; T1 post (9 weeks); T2 3 months; T3 12 months	*N* randomized/analyzed: Randomized *N *= 76 (38/38). Analyzed: T0 *n *= 24 EG, *n *= 29 CG; T1 *n *= 18 EG, *n *= 26 CG; T2 *n *= 17 EG, *n *= 24 CG; T3 *n *= 17 EG, *n *= 20 CG Population: adults with IBD (Crohn's/UC) Age/mean: NR Sex (M/F): EG 11/13; CG 13/16	Clinical outcomes: psychological distress (DASS‐21); IBD symptom perception (IBDSS); health‐related QoL (IBDQ‐UK) Process outcomes: self‐compassion (SCS: self‐compassionate and self‐critical attitude); psychological flexibility (CompACT); chronic illness‐related shame (CISS); functional impairment (WSAS); general QoL (EUROHIS‐QOL‐8)	No significant omnibus Time×group effects for self‐compassion; some evidence for anxiety (group *p *= 0.044; time×group *p *= 0.050) and stress time effect (*p *= 0.040). Self‐compassionate attitude showed a within‐EG improvement from baseline to 3‐month follow‐up.	Effect size (or *p* value): self‐compassionate attitude (within EG): T0→T2 *p *= 0.045, SMD =0.69. Omnibus: self‐critical attitude group *p *= 0.004; psychological flexibility group *p *= 0.003; anxiety group *p *= 0.044; time × group *p*=0.050 Certainty of evidence/RoB: RoB 2 (Overall: Some concerns)	Adverse events: NR Dropouts: high attrition across timepoints (see analyzed *n* above)	ACT intervention: LIFEwithIBD (ACT + compassion‐based group program) Delivery mode: in‐person group; 9‐week program Comparator: control/TAU (NR: full description)	Self‐compassion: SCS (self‐compassionate/self‐critical attitude); no significant time × group; within EG self‐compassionate attitude ↑ at T2 (*p *= 0.045) Key ACT processes: Psychological flexibility (CompACT) assessed; group effect *p *= 0.003 (EG poorer). Shame (CISS) assessed (omnibus NR for time × group)
Author: Köhle et al. [28] Year: 2017 Location/country: The Netherlands	To explore user experiences with a web‐based self‐help intervention for partners of cancer patients based on ACT and self‐compassion.	Design: qualitative interview study (in‐depth interviews) Arms: NR (participants had used the intervention) Dose and period: web‐based self‐help with 6 lessons (tunneling); weekly feedback messages (counselor) Timepoints: post‐intervention interviews (timing NR)	*N* randomized/analyzed: NR; *N *= 14 interviewed Population: partners of cancer patients (from ongoing RCT) Age/mean: 55.3 ± 9.3 years (range 34–68) Sex (M/F): 3/11	Outcomes: qualitative themes (user experiences; perceived changes)	Participants described gaining insight and skills consistent with psychological flexibility and self‐compassion (e.g., acceptance, cognitive defusion, values/committed action), and perceived the intervention as useful.	Effect size (or *p* value): NR (qualitative study) Certainty of evidence/RoB: ROBINS‐I (Overall: no information)	Adverse events: NR Dropouts: NR	ACT intervention: “Hold on, for each other” web‐based self‐help (ACT + self‐compassion) Delivery mode: Online (6 lessons) + weekly counselor feedback messages Comparator: none (qualitative)	Self‐compassion: discussed qualitatively (no quantitative measure reported) Key processes: acceptance, defusion, values and committed action described as perceived changes (qualitative)
Author: Köhle et al. [38] Year: 2021 Location/country: The Netherlands	To evaluate two versions of a web‐based ACT + self‐compassion self‐help intervention (personal vs. automated feedback) for partners of cancer patients, compared with waitlist.	Design: randomized controlled trial (3‐arm) Arms: personal feedback (PF) vs. automated feedback (AF) vs. waitlist (WL) Dose and period: web‐based self‐help, 6 lessons; recommended 1–1.5 h/week (as reported) Timepoints: T0 baseline; T1 post (3 months); T2 follow‐up (6 months; PF/AF only)	*N* randomized/analyzed: Randomized *N *= 203 (PF 67; AF 70; WL 66) Population: partners of cancer patients/survivors Age/mean: 55.89 ± 10.72 years (range 27–82) Sex (M/F): 60/143	Clinical outcomes: psychological distress (HADS) and other partner outcomes (NR: full list) Process outcomes: self‐compassion; psychological flexibility; resilience; coping/communication styles (protective buffering/overprotection)	No significant improvements in primary/secondary outcomes vs. waitlist at post. PF improved psychological flexibility and resilience and reduced overprotection vs. WL; AF reduced overprotection/protective buffering vs. WL. Self‐compassion did not significantly improve vs. WL.	Effect size (or *p* value): PF vs. WL: psychological flexibility *d *= 0.49; resilience *d *= 0.12; overprotection *d *= 0.25. Self‐compassion: PF‐WL F = 2.22 (*p *= 0.139); AF‐WL F = 2.48 (*p *= 0.119); PF‐AF F = 3.16 (*p *= 0.045) Certainty of evidence/RoB: RoB 2 (Overall: some concerns)	Adverse events: NR Dropouts: NR (adherence reported; proportion completing intervention 69.2%)	ACT intervention: web‐based self‐help (ACT + self‐compassion) with 6 lessons Delivery mode: online; PF via weekly counselor e‐mail feedback; AF via automated feedback Comparator: waitlist control	Self‐compassion: measured; no significant change vs. WL at post (*p *= 0.139–0.119) Key ACT processes: psychological flexibility improved in PF vs. WL (*d* = 0.49); communication coping (overprotection/protective buffering) improved
Author: Ong et al. [39] Year: 2019 Location/country: United States	To examine psychological inflexibility and self‐compassion as mediators/moderators of outcomes following ACT for clinical perfectionism.	Design: randomized controlled trial (parallel groups) Arms: ACT (10 sessions) vs. waitlist (14 weeks) Dose and period: 10 weekly ACT sessions Timepoints: pre, post, 1‐month follow‐up	*N* randomized/analyzed: Randomized *N *= 53; post‐treatment *N *= 39; follow‐up *N *= 31 Population: adults with clinical perfectionism Age/mean: 25.4 ± 12.3 years Sex (M/F): NR/73.6% female (as reported)	Clinical outcomes: perfectionism dimensions (FMPS subscales); quality of life; symptom distress/functional impairment; valued action Process outcomes: psychological inflexibility (AAQ‐II); self‐compassion (SCS)	ACT‐related decreases in psychological inflexibility mediated increases in quality of life; increases in self‐compassion mediated reductions in concern over mistakes. Baseline psychological inflexibility and self‐compassion moderated some outcomes.	Effect size (or *p* value): Condition→AAQ‐II (a path) *p *= 0.010; AAQ‐II→quality of life (b path) *p *= 0.028. Self‐compassion→concern over mistakes (b path) *p *= 0.023 Certainty of evidence/RoB: RoB 2 (Overall: high)	Adverse events: NR Dropouts: Completed post: 39/49 eligible; follow‐up: 31/49 eligible (as reported)	ACT intervention: Manualized ACT targeting perfectionism (did not explicitly target self‐compassion) Delivery mode: in‐person weekly sessions (10) Comparator: waitlist	Self‐compassion: SCS; increased and mediated reductions in concern over mistakes (*p *= 0.023) Key ACT processes: Psychological inflexibility (AAQ‐II) decreased; mediated quality of life improvements (*p *= 0.028)
Author: Yadavaia et al. [25] Year: 2014 Location/country: United States	To test an ACT‐based workshop aimed at increasing self‐compassion in distressed undergraduates, and examine mediation by psychological flexibility and moderation by trauma history.	Design: randomized controlled trial (parallel groups) Arms: ACT workshop vs. waitlist Dose and period: 6‐h group workshop Timepoints: pre, post, follow‐up (~2 months)	*N* randomized/analyzed: Randomized *N *= 73 (ACT 30; WL 43); post *n *= 28/39; follow‐up *n *= 28/40 Population: undergraduates with low self‐compassion and elevated distress Age/mean: ACT 19.90 ± 4.05; WL 20.83 ± 4.61 Sex (M/F): ACT 8/22; WL 13/30	Clinical outcomes: general health (GHQ); depression/anxiety/stress (DASS‐21) Process outcomes: self‐compassion (SCS); psychological flexibility (AAQ‐II)	Compared with waitlist, ACT led to higher self‐compassion and better general health at post and follow‐up; psychological flexibility mediated effects. Trauma history moderated symptom outcomes (greater trauma associated with larger improvements).	Effect size (or *p* value): between‐group post/follow‐up *t*‐tests: Self‐compassion *p *= 0.00 (post; follow‐up); GHQ *p *= 0.00 (post; follow‐up); Depression *p *= 0.01 (post); Stress *p *= 0.04 (follow‐up); AAQ‐II *p *= 0.01 (follow‐up). Time × condition for self‐compassion: *F*(2,106.8) = 3.69, *p *= 0.03 (effect size = 0.45) Certainty of evidence/RoB: RoB 2 (Overall: some concerns)	Adverse events: NR Dropouts: Post: ACT 2; WL 4. Follow‐up: ACT 2; WL 3 (based on reported *n*)	ACT intervention: ACT‐based self‐compassion workshop Delivery mode: in‐person group, single 6‐h workshop Comparator: waitlist	Self‐compassion: SCS; higher in ACT vs. WL at post and follow‐up (*p *= 0.00) Key ACT processes: Psychological flexibility (AAQ‐II) improved at follow‐up (*p *= 0.01) and mediated changes
Author: Hill et al. [40] Year: 2020 Location/country: United States	To evaluate feasibility and outcomes of compassion‐focused ACT for restrictive eating and body‐checking in women using a multiple baseline single‐case design.	Design: nonconcurrent multiple baseline across participants (single‐case) Arms: baseline vs. treatment phase (no parallel comparator) Dose and period: 10 individual sessions; 50 min each Timepoints: daily self‐monitoring; midpoint, post‐treatment, 3‐month follow‐up	*N* randomized/analyzed: NR; *N *= 3 Population: women with restrictive eating and body‐checking concerns Age/mean: 19–24 years (P1 = 23; P2 = 24; P3 = 19) Sex (M/F): 0/3	Clinical outcomes: daily ED target behaviors; global ED pathology (EDE‐Q); general distress (GHQ‐12); weight Process outcomes: self‐compassion (SCS‐SF); body image flexibility (BI‐AAQ)	Visual inspection suggested decreases in target ED behaviors and improvements in valued behavior, self‐compassion, and body image flexibility across participants, with reductions in ED pathology and distress.	Effect size (or *p* value): NR (single‐case; visual/idiographic analyses reported) Certainty of evidence/RoB: ROBINS‐I (Overall: serious)	Adverse events: NR Dropouts: NR	ACT intervention: Compassion‐focused ACT (outpatient) Delivery mode: In‐person individual therapy Comparator: baseline phase	Self‐compassion: SCS‐SF; improved across participants (quantitative values NR) Key ACT processes: Body image flexibility (BI‐AAQ) improved; values‐based action tracked
Author: Kılıç et al. [27] Year: 2022 Location/country: United Kingdom	To examine shared and unique longitudinal contributions of self‐compassion and psychological inflexibility to distress and quality of life in adults with Type 2 diabetes over 12 months.	Design: longitudinal observational study (online assessments) Arms: none Dose and period: 12‐month follow‐up Timepoints: T1 baseline; T2 6 months; T3 12 months	*N* randomized/analyzed: NR; *N *= 173 baseline; *N *= 82 (6 months); *N *= 52 (12 months) Population: UK adults with Type 2 diabetes Age/mean: 58.3 ± 11.80 years Sex (M/F): 67/104 (sex NR for 2 participants)	Clinical outcomes: depression (PHQ‐8); anxiety (GAD‐7); diabetes distress (PAID); QoL (EQ‐5D‐3L VAS) Process outcomes: self‐compassion (SCS); psychological inflexibility (AAQ‐II)	Self‐compassion and psychological inflexibility were strongly negatively correlated across time. In regressions controlling baseline outcomes, psychological inflexibility uniquely predicted later depression/anxiety and QoL; self‐compassion did not uniquely predict outcomes.	Effect size (or *p* value): Correlation (baseline): AAQ‐II vs. SCS *r *= −0.69 (*p*< 0.01). Regression: psychological inflexibility predicted depression at 6 months (*B *= 0.23, *p *= 0.02) and anxiety at 6 months (*B *= 0.17, *p *= 0.02) and 12 months (*B *= 0.23, *p *= 0.002), and QoL at 12 months (*B* = −0.81, *p *= 0.04) Certainty of evidence/RoB: ROBINS‐I (Overall: serious)	Adverse events: NR Dropouts: attrition to 82 (T2) and 52 (T3) from 173 baseline	ACT intervention: No ACT intervention (observational study) Delivery mode: NR Comparator: NR	Self‐compassion: SCS; correlated with distress but not a unique longitudinal predictor in regression models Key ACT processes: Psychological inflexibility (AAQ‐II) uniquely predicted later distress and QoL

Abbreviations: AAQ‐II, acceptance and action questionnaire‐II; ACT, acceptance and commitment therapy; AF, automated feedback; BI‐AAQ, body image–acceptance and action questionnaire; BPI‐IS, brief pain inventory—interference scale; CAQ‐8, committed action questionnaire—8‐item; CFQ‐7, cognitive fusion questionnaire—7‐item; CISS, chronic illness‐related shame scale; CompACT, comprehensive assessment of ACT processes; CPAQ‐8, chronic pain acceptance questionnaire—8‐item; d, Cohen's d effect size; DASS‐21, depression anxiety stress scales—21‐item; ED, eating disorder; EDE‐Q, eating disorder examination questionnaire; EG, experimental group; EQ‐5D‐3L, EuroQol 5‐dimension 3‐level; ERQ, emotion regulation questionnaire; EUROHIS‐QOL‐8, EUROHIS quality of life—8‐item index; GAD‐7, generalized anxiety disorder—7‐item; GHQ‐12, General Health Questionnaire—12‐item; HADS, hospital anxiety and depression scale; IBD, inflammatory bowel disease; IBDQ‐UK, inflammatory bowel disease questionnaire—UK; IBDSS, IBD symptom scale; NR, not reported; PAID, problem areas in diabetes; PF, personal feedback; PHQ‐8/PHQ‐9, patient health questionnaire—8/9‐item; QoL, quality of life; RCT, randomized controlled trial; RFT, Relational Frame Theory; RoB, risk of bias; ROBINS‐I, risk of bias in non‐randomized studies of interventions; SCS, self‐compassion scale; SCS‐SF, self‐compassion scale—short form; SD, standard deviation; SEQ, self experiences questionnaire; SMD, standardized mean difference; SSCI‐8, stigma scale for chronic illness—8‐item; TAU, treatment as usual; UC, ulcerative colitis; VAS, visual analogue scale; WL, waitlist; WSAS, work and social adjustment scale; *η*², eta squared effect size.

Self‐compassion was most frequently measured using the Self‐Compassion Scale (SCS) or a validated short form. Some studies reported total scores, while others reported mean item scores, limiting direct comparability across studies. Follow‐up durations ranged from immediate post‐intervention to 12 months, and comparators included waitlists, treatment‐as‐usual, and alternative forms of guidance or feedback within the same digital intervention. Given substantial heterogeneity in populations, interventions, and reporting metrics, and limited availability of compatible effect estimates, meta‐analysis was not conducted. Instead, results were synthesized narratively, with effect sizes and uncertainty reported where provided or calculable from available summary data. Outcome domains, measurement instruments, and the availability of effect estimates and uncertainty metrics across included studies are summarized in Table 2.

**Table 2:** Outcome domains and key findings across included studies.

Study (design; evidence stream)	Self‐compassion (primary)	Self‐criticism/related constructs	Emotional well‐being/psychological distress	ACT‐relevant process measures and other outcomes
Yadavaia et al., 2014 [25] (RCT; intervention)	SCS. Greater improvement vs. waitlist (time × group *p* < 0.001). Model‐based change in ACT arm: 4.82 (95% CI 3.66–5.99); large within‐group change (*d* = 1.54).	Not reported as distinct outcome.	General distress and depressive symptoms decreased more in ACT than waitlist at follow‐up; anxiety between‐group differences not statistically significant.	AAQ‐II (psychological flexibility) improved; change in flexibility statistically linked to change in self‐compassion (mediation reported).
Ong et al., 2019 [39] (RCT; intervention)	SCS. ACT predicted higher self‐compassion at post‐treatment (*B* = 3.17, SE 0.74; *p* < 0.001). Between‐group SMD not reported.	Perfectionism facet (concern over mistakes) decreased in lagged models following higher self‐compassion (*B* = −0.48, SE 0.20; *p* < 0.05).	Distress outcomes reported; effect sizes for self‐compassion not provided in primary report.	Process variables analyzed in time‐lagged models (mechanistic framing); detailed effect metrics vary by model.
Köhle et al., 2021 [38] (RCT; intervention)	SCS (model‐based marginal means). No significant condition × time contrasts vs. waitlist at post; PF vs. AF trajectories differed (*p* = 0.045). Effect size not reported.	Not reported as distinct outcome.	Positive mental health reported; format‐specific trajectories differed at later follow‐up (condition × time *p* = 0.04 for positive mental health in PF vs. AF comparisons).	Psychological flexibility and coping‐related outcomes reported; some format contrasts significant at post (e.g., flexibility/resilience in PF vs. AF contrasts).
Ferreira et al., 2024 [29] (RCT; intervention)	Self‐compassionate attitude. No clear between‐group difference at post. Within‐group contrast in intervention arm at follow‐up: SMD = 0.69 (95% CI 0.002–1.38).	Self‐critical attitude. Post‐treatment means similar between groups; authors reported larger time‐specific contrasts at later follow‐up (SMD up to 1.15, 95% CI 0.42–1.87).	Depression/anxiety/stress (DASS) and QoL outcomes reported; post‐treatment group differences generally not significant after baseline adjustment.	IBD symptom perception and disease activity indices reported; some time effects observed; ACT‐consistent processes reported variably.
Fattahi et al., 2023/2025 [30] (RCT; intervention)	Self‐compassion increased more in ACT than control (time × group *F* = 10.87, *p* = 0.01; *η*p²=0.24).	Not reported as distinct outcome.	Depression, anxiety, and stress improved with effects favoring ACT over time; emotion regulation indices also improved.	Emotion regulation (reappraisal/suppression) reported; multiple outcomes analyzed longitudinally.
Anderson et al., 2024 [26] (pre–post; intervention service evaluation)	SCS‐SF. Small pre–post improvement (*d* = 0.21). No control group.	Not reported as distinct outcome.	Depression/anxiety and pain‐related outcomes improved pre–post (service evaluation).	ACT process measures reported (e.g., self‐as‐context); pain interference decreased with larger effects than self‐compassion.
Hill et al., 2020 [40] (single‐case multiple baseline; intervention)	SCS. Increases observed for all participants from baseline to post and follow‐up (descriptive; no standardized effect).	Not reported as distinct outcome.	General distress (GHQ) improved for some participants; outcomes reported at individual level.	Body image flexibility and related measures reported; single‐case design limits generalizability.
Boland et al., 2021 [41] (laboratory experiment; contextual/process)	State self‐compassion increased across repeated stages (within‐subject *η*p²=0.48); small interaction effects reported.	Not reported as distinct outcome.	Emotional discomfort decreased over stages (within‐subject effect reported); clinical outcomes not assessed.	State cognitive fusion decreased over stages (within‐subject effect reported); perspective‐taking manipulation examined.
Kılıç et al., 2022 [27] (12‐month observational; contextual/process)	SCS baseline levels reported; self‐compassion correlated with psychological inflexibility (*r* = −0.69).	Not reported as distinct outcome.	Distress and QoL outcomes modeled longitudinally; self‐compassion contributed inconsistently once inflexibility and covariates were included.	AAQ‐II (psychological inflexibility) predicted later distress outcomes in several models.
Köhle et al., 2017 [28] (qualitative; contextual/process)	No quantitative self‐compassion endpoint; themes consistent with more compassionate self‐responding described.	Qualitative themes related to self‐blame/self‐responding reported.	Caregiver burden and coping experiences described qualitatively; no quantitative well‐being effect estimates.	ACT‐consistent processes (acceptance/values‐based coping) described in participant narratives.

*Note:* This table summarizes outcome domains, measures, and the availability of effect estimates/uncertainty for the included intervention and contextual/process studies. Results are reported as presented in the primary studies (meta‐analysis not performed).

Intervention studies estimate changes following ACT or ACT‐based interventions. Contextual/process studies describe associations or experimental effects without delivering ACT; these findings are not interpreted as treatment efficacy.

Abbreviations: AAQ‐II, acceptance and action questionnaire‐II; ACT, acceptance and commitment therapy; AF, automated feedback; DASS, depression anxiety stress scales; GHQ, general health questionnaire; IBD, inflammatory bowel disease; PF, personal feedback; QoL, quality of life; RCT, randomized controlled trial; SCS, self‐compassion scale; SCS‐SF, self‐compassion scale–short form; SMD, standardized mean difference.

### 3.4 Primary Outcome: Self‐Compassion

Self‐compassion was assessed in all included studies, most often using the Self‐Compassion Scale (SCS) in its full or short form, although studies differed in whether they reported total scores, mean scores, or model‐based marginal means [25, 26, 28, 29, 30, 38, 39, 40, 41]. In controlled trials, self‐compassion typically improved over time in intervention arms, yet the extent of separation from comparator conditions varied across settings and follow‐up windows [25, 29, 30, 38, 39]. Where quantification was available, findings ranged from minimal between‐group differences at post‐treatment to large improvements in selected samples.

The clearest controlled signal came from a brief ACT workshop trial, in which model‐based estimates indicated a larger pre‐to‐follow‐up increase in self‐compassion for the ACT arm than for the waitlist, with the ACT change estimate exceeding the control estimate and the ACT within‐group standardized change reported as large [25]. In the trial targeting clinical perfectionism, self‐compassion was examined as a time‐lagged mediator, and assignment to ACT predicted higher self‐compassion at post‐treatment (*B* = 3.17, SE 0.74; *p* < 0.001), although raw between‐group self‐compassion means and standardized between‐group effect sizes were not reported [39].

Digital and chronic disease trials provided more mixed controlled evidence. In the web‐based intervention for partners of patients with cancer, intention‐to‐treat models did not show a statistically significant condition‐by‐time contrast for self‐compassion relative to waitlist over baseline to post‐treatment, while comparisons between feedback modalities suggested different trajectories at later follow‐up [38]. In the inflammatory bowel disease trial, adjusted analyses did not indicate clear post‐treatment group differences, and a within‐group contrast in the intervention arm at follow‐up was reported with a moderate standardized change and a wide confidence interval (SMD = 0.69; 95% CI 0.002–1.38) [29]. In the cardiovascular disease trial, self‐compassion increased markedly in the ACT arm relative to control over the study period, with a statistically significant time‐by‐group effect reported and large between‐group separation described by the authors [30].

Nonrandomized, experimental, and qualitative evidence were broadly consistent with within‐person increases in self‐compassion, while offering limited support for causal attribution. A large pre–post evaluation of an ACT‐based chronic pain program reported a small improvement in self‐compassion (*d* = 0.21) [26]. A multiple‐baseline case series reported increases in self‐compassion for each participant from baseline to post‐treatment that were largely retained at follow‐up, but without standardized effect estimates [40]. A laboratory experiment reported a large within‐subject increase in state self‐compassion over repeated stages (*η*p² = 0.48) and smaller group‐dependent patterns [41]. A longitudinal observational study in type 2 diabetes reported a strong negative association between self‐compassion and psychological inflexibility (*r* = −0.69), and models in that study indicated that self‐compassion contributed inconsistently once psychological inflexibility and covariates were considered [27]. A qualitative caregiver study described perceived increases in compassionate self‐responding during and after participation, without quantitative self‐compassion endpoints [28].

### 3.5 Secondary Outcomes and ACT‐Consistent Process Measures: Self‐Criticism, Emotional Well‐Being, and Related Contextual Outcomes

Self‐criticism was explicitly operationalized as a self‐critical attitude measure in the inflammatory bowel disease trial, which provided the most direct evidence on this outcome [29]. Post‐treatment scores were similar between groups, with an unadjusted mean difference close to zero and a wide confidence interval, indicating little separation at that time point. Time‐specific contrasts reported by the authors suggested larger between‐group separation at later follow‐ups, with standardized differences ranging from moderate to large depending on the assessment point (up to SMD = 1.15; 95% CI 0.42–1.87) [29]. Other included studies did not consistently report self‐criticism as a distinct endpoint, which limited synthesis beyond this trial.

Emotional well‐being was operationalized heterogeneously across studies and was therefore synthesized as a broad secondary domain rather than as a single pooled endpoint. Measures included positive mental health, psychological distress, depression, anxiety, stress, emotion regulation, quality of life, resilience, and selected ACT‐consistent process measures such as psychological flexibility, psychological inflexibility, cognitive fusion, perspective‐taking, and body image flexibility. ACT‐consistent process measures are reported here only when they were assessed alongside the review outcomes and are used to support interpretation of possible mechanisms rather than to establish additional efficacy endpoints.

In the ACT workshop trial, general psychological distress decreased more in the ACT arm than in the waitlist arm, and depressive symptoms showed clearer between‐group separation than anxiety symptoms at post‐treatment and follow‐up [25]. Stress also decreased in the ACT arm with evidence of between‐group differences at follow‐up, while anxiety outcomes showed less consistent between‐group separation despite within‐arm reductions in the intervention condition [25]. The same trial reported improvements in psychological flexibility (AAQ‐II) over time, with a between‐group difference reported at follow‐up [25].

In the cardiovascular disease trial, depression, anxiety, stress, emotion regulation indices, and self‐compassion all showed improvement patterns favoring ACT, with statistically significant time‐by‐group effects reported for multiple outcomes and large between‐group separation described by the authors [30]. In the cancer partner trial, intention‐to‐treat analyses did not show a statistically significant intervention‐by‐time effect on psychological distress at post‐treatment relative to waitlist, whereas per‐protocol analyses reported significant effects for psychological distress with small‐to‐moderate effect sizes [38]. Longer follow‐up comparisons between the two active formats also indicated statistically significant condition‐by‐time effects for positive mental health and self‐compassion, while process measures and related outcomes differed by format in some contrasts, including psychological flexibility and resilience [38].

In the inflammatory bowel disease trial, baseline‐adjusted analyses did not indicate clear post‐treatment group differences in distress measures, while longitudinal modeling suggested time effects for some outcomes without consistent group‐by‐time interactions. A disease activity index differed at a follow‐up time point, and acceptability indicators were high among completers, including universal endorsement of recommending the intervention to another person with inflammatory bowel disease (18/18) [29].

Nonrandomized evidence further reported changes in distress and ACT‐consistent processes in designs without randomized comparators. In the ACT‐based chronic pain service evaluation, pre–post improvements were reported across several outcomes, with larger effect sizes for pain interference and depressive symptoms than for self‐compassion [26]. In the multiple‐baseline case series, general distress decreased substantially for two participants from baseline to follow‐up, while one participant showed a smaller change, alongside improvements in body image flexibility [40]. In the experimental perspective‐taking study, emotional discomfort and state cognitive fusion decreased with large within‐subject effects over repeated stages, while between‐group differences were smaller and less consistent [41].

Taken together, findings suggested that self‐compassion can increase following ACT and ACT‐consistent process interventions, yet the magnitude and durability of between‐group differences varied widely and were influenced by intervention format, comparator condition, risk of bias, and the heterogeneous operationalization of emotional well‐being across studies.

## 4 Discussion

This systematic review synthesized evidence from 10 studies examining self‐compassion outcomes in the context of Acceptance and Commitment Therapy (ACT) across diverse mental and physical health contexts. The overall pattern suggests that self‐compassion often improves during ACT‐based interventions, but the strength of evidence for ACT producing greater improvements than control conditions is mixed. Among RCTs, a brief, intensive ACT workshop in a selected distressed student sample showed a robust between‐group signal on self‐compassion [25], while other RCTs showed smaller, inconsistent, or time‐limited differences [29, 30, 38]. A trial focused on clinical perfectionism identified self‐compassion as a plausible process variable in lagged mediation models [39], yet high risk of bias and attrition reduce confidence in precise effect estimates. Nonrandomised and experimental findings were broadly consistent with ACT‐related processes supporting compassionate self‐responding [26, 40, 41], but these designs cannot establish causality.

### 4.1 Interpreting a Heterogeneous Signal

The variability in findings likely reflects meaningful differences in how strongly interventions activated compassion‐relevant processes, as well as methodological factors. ACT does not always explicitly teach compassion skills, and self‐compassion may increase indirectly through psychological flexibility processes such as acceptance, defusion, and perspective‐taking. Evidence from chronic pain research emphasizes psychological flexibility as a core model for adjustment and a clinically relevant target across interventions [42, 43]. Observational data also support that psychological flexibility and self‐compassion are related yet distinct constructs, each contributing to emotional well‐being [24]. From this perspective, ACT may produce increases in self‐compassion when (i) interventions include explicit compassion exercises, metaphors, or therapist modeling of compassionate responding, (ii) baseline self‐compassion is low, leaving greater room for change, and (iii) treatment intensity is sufficient to consolidate new self‐responding patterns.

Comparator choice also matters. Waitlist controls may overestimate intervention effects relative to more active comparators. In addition, follow‐up timing may shape conclusions. The cardiovascular disease trial suggested a marked post‐treatment increase in self‐compassion that narrowed by follow‐up [30], raising the possibility that self‐compassion gains may be less stable without continued practice or booster inputs in some populations. In contrast, the brief ACT workshop study reported sustained gains in self‐compassion to follow‐up [25], although this trial recruited participants based on low self‐compassion and distress, which may inflate observed responsiveness.

### 4.2 What This Review Adds Beyond Existing Syntheses

Existing ACT syntheses have primarily evaluated symptom outcomes (e.g., anxiety, depression, and general distress), often across broad clinical samples, and typically do not treat self‐compassion as a primary endpoint. This gap matters because self‐compassion has become a prominent clinical target in its own right, linked to resilience, emotion regulation, and adaptive coping across mental and physical health conditions. Separate syntheses of self‐compassion interventions show benefits for distress and well‐being, yet these reviews often pool diverse compassion‐focused programs and do not isolate ACT‐specific contexts. A systematic review of ACT in chronic disease and long‐term conditions highlights promising effects across psychological and functional outcomes, but also emphasizes variability in intervention delivery and outcome selection that complicates clear conclusions about specific mechanisms [44]. Similarly, ACT‐focused reviews in chronic pain indicate small‐to‐moderate improvements across outcomes but note methodological heterogeneity and reliance on self‐report measures [45, 46].

By focusing specifically on self‐compassion outcomes within ACT and ACT‐consistent interventions, the present review complements these broader syntheses and clarifies that improvements in self‐compassion are plausible but not uniform. Importantly, the current evidence does not support strong claims that ACT reliably produces large between‐group advantages in self‐compassion across conditions, nor that adding self‐compassion content is always necessary. Instead, findings suggest a nuanced position: self‐compassion can improve during ACT, but whether it improves beyond comparator conditions may depend on treatment content, population, and study design.

### 4.3 Implications for Chronic Disease and Rehabilitation Contexts

Several included studies were conducted in chronic disease and caregiving contexts (inflammatory bowel disease, cardiovascular disease, and cancer caregiving), where persistent symptoms, uncertainty, and role strain commonly increase self‐criticism and distress. The broader evidence base supports ACT as a viable approach across chronic health conditions [44], and ACT has established relevance in chronic pain management [45, 46]. Systematic evidence also indicates that self‐compassion‐oriented approaches may be beneficial for people living with chronic physical health conditions [47], and specific syntheses focusing on chronic pain suggest that higher self‐compassion is associated with better functioning and lower distress [48]. These converging lines of evidence make self‐compassion a clinically meaningful outcome for rehabilitation and long‐term condition care, even when it is not the sole target.

For neurorehabilitation, psychological adjustment to neurological disability often involves self‐judgment, loss‐related distress, and barriers to values‐based re‐engagement. Although neurorehabilitation populations were not directly represented among the included intervention trials in this review, adjacent evidence suggests potential relevance. For example, ACT has been tested in multiple sclerosis (MS), with a small RCT indicating beneficial effects on depression and quality of life [49], and a systematic review and meta‐analysis suggesting that ACT may improve psychological outcomes in MS [50]. In acquired brain injury contexts, compassion‐focused approaches have been explored as feasible and potentially helpful, though evidence remains preliminary [51]. Together, these studies support the plausibility of integrating ACT and compassion‐focused components in neurorehabilitation pathways, particularly where self‐criticism and shame impede participation, persistence, and re‐engagement with valued activities.

At the same time, translation to neurorehabilitation requires caution. The review's intervention evidence on self‐compassion is derived from heterogeneous populations, and the quality of evidence varies. Neurorehabilitation trials that explicitly measure self‐compassion, self‐criticism, and psychological flexibility as targets and mechanisms are needed before making strong clinical recommendations specific to neurological conditions.

### 4.4 Methodological Implications and Priorities for Future Research

A key limitation across the included evidence is inconsistent outcome reporting and limited availability of comparable effect estimates. Self‐compassion was assessed using different scoring formats (total scores vs. mean scores; full scale vs. short forms), and several studies did not report sufficient statistics to support robust effect estimation. These reporting issues precluded meta‐analysis and constrained synthesis to a narrative approach.

Future trials should aim to improve interpretability by (i) pre‐registering self‐compassion outcomes and analysis plans, (ii) reporting complete outcome data with effect sizes and confidence intervals, (iii) including active comparators where feasible, and (iv) standardizing follow‐up windows to clarify the durability of change. Studies designed to test mechanisms should incorporate mediator models that evaluate whether changes in psychological flexibility precede and predict changes in self‐compassion, or vice versa, using time‐lagged designs with an adequate sample size.

There is also a practical intervention‐development question that remains unresolved: does ACT that explicitly incorporates compassion skills produce greater gains in self‐compassion than standard ACT? Hybrid approaches already exist in the broader chronic pain literature, including internet‐delivered programs combining ACT and compassion‐focused therapy components [52], and targeted self‐compassion interventions that can be delivered online [53, 54]. These formats align with rehabilitation service constraints where scalability and adherence support are critical. Conceptually, this direction is consistent with a process‐based approach in which clinicians select and intensify techniques that best target the maintaining processes relevant to the individual [55]. However, robust head‐to‐head trials are needed to determine whether adding explicit compassion‐focused modules meaningfully improves outcomes beyond standard ACT processes.

### 4.5 Strengths and Limitations of This Review

This review followed PRISMA 2020 guidance and included duplicate screening with adjudication and assessment of interrater agreement. Multiple databases were searched and risk of bias was evaluated using contemporary tools appropriate to study design. The synthesis prioritized transparent reporting of effect estimates and uncertainty when available or calculable.

Limitations include substantial heterogeneity across included studies, limited ability to pool data quantitatively, and a predominance of self‐report measures. A portion of the evidence base came from nonrandomised designs judged at serious risk of bias, which limits confidence in causal interpretations. Publication and reporting biases cannot be ruled out, and restrictions to English‐language publications may have excluded some relevant evidence.

## 5 Conclusions

Across 10 heterogeneous studies, self‐compassion often improved during ACT‐based interventions, but evidence for consistent between‐group superiority over comparator conditions was mixed and was limited by risk of bias and variable reporting. The strongest RCT evidence suggested that intensive ACT interventions can produce meaningful gains in self‐compassion in selected samples, while other trials showed smaller or transient differences. Nonrandomised and experimental findings supported the plausibility that ACT‐consistent processes may foster compassionate self‐responding, yet causal inference remains limited outside randomized designs. Future research should prioritize adequately powered, well‐reported trials that standardize self‐compassion outcomes and test whether explicit compassion‐focused components add benefit beyond standard ACT, particularly in rehabilitation‐relevant populations where self‐criticism and adjustment difficulties are common.

## Author Contributions

**Andrea Calderone:** conceptualization. **Desirèe Latella:** methodology, writing – original draft. **Giulia Marafioti:** conceptualization. **Caterina Formica:** visualization. **Angela Foti:** investigation. **Elvira la Fauci:** investigation. **Giuseppa Filippello:** validation.

## Ethics Statement

The authors have nothing to report.

## Conflicts of Interest

The authors declare no conflicts of interest.

## Transparency Statement

The corresponding author, Caterina Formica, affirms that this manuscript is an honest, accurate, and transparent account of the study being reported; that no important aspects of the study have been omitted; and that any discrepancies from the study as planned (and, if relevant, registered) have been explained.

## Supporting information

Supporting File 1:

Supporting File 2:

## Data Availability

Data extracted from included studies are reported in the manuscript and its supplementary materials. The underlying data extraction files and analytic worksheets used for the narrative synthesis are available from the corresponding author upon reasonable request.
