# Supplementary material for: Effects of Acceptance and Commitment Therapy on Self‐Compassion, Self‐Criticism, and Emotional Well‐Being in Adults With Mental Health Concerns: A Systematic Review
Source: Health Sci Rep. 2026 May 25;9(6):e72525. doi: 10.1002/hsr2.72525 (PMC13240463; doi:10.1002/hsr2.72525)
Supplement: Supplementary file 1 — Supporting File 1: [file HSR2-9-e72525-s001.docx]

**Supplementary Table S1. Instruments for assessing self-compassion, compassion, and related constructs**

This table provides commonly used self-report instruments relevant to ACT and self-compassion research. The table is intended to support consistent operationalization and reporting across studies.

| **Instrument** | **Construct focus** | **Domains and format** | **Scoring and reporting** | **Practical notes and key references** |
| --- | --- | --- | --- | --- |
| Self-Compassion Scale (SCS) | Trait self-compassion (self-kindness, common humanity, mindfulness; and reduced self-judgment, isolation, over-identification) | 26 items; 5-point Likert; 6 subscales and global score | Mean score (after reverse scoring negative items); report global and/or subscales; higher = greater self-compassion | Most widely used self-compassion measure; suitable for baseline assessment and intervention change. Refs: Neff 2003; French validation. [1,2] |
| Self-Compassion Scale-Short Form (SCS-SF) | Trait self-compassion (brief global assessment) | 12 items; 5-point Likert; mirrors 6 facets of the long form | Mean global score recommended; higher = greater self-compassion | Preferred when questionnaire burden is high; correlates strongly with long form. [3] |
| State Self-Compassion Scale (SSCS) | State self-compassion in response to a specific negative event | Long form 18 items; short form 6 items; 5-point Likert | Mean total (and 6 components for long form); higher = greater state self-compassion | Useful for session-level or experimental change where trait measures may be less sensitive. [4] |
| Brief Self-Compassion Inventory (BSCI) | Unidimensional brief self-compassion measure | 5 items; brief screening format | Sum or mean of items; higher = greater self-compassion | Designed for clinical settings requiring very brief assessment; initial validation in adults with cancer. [5] |
| Self-Compassion Scale for Youth (SCS-Y) | Trait self-compassion for early adolescents | 17 items; multidimensional; age-appropriate wording | General self-compassion score and optional subscales; higher = greater self-compassion | Useful when adolescent samples are included; supports developmental measurement. [6] |
| Self-Compassion and Self-Criticism Scales (SCCS) | Situational self-compassion and self-criticism in response to scenarios | Scenario-based responses (multiple hypothetical situations) | Separate subscale scores for self-compassion and self-criticism; higher = more of each construct | Designed to capture state-like responding and sensitivity to change under challenge. [7] |
| Self-Compassionate Reactions Inventory (SCRI) | Scenario-based self-compassionate responding to hardships | 8 hypothetical hardships with response options; versioned adaptations exist | Higher scores indicate greater selection of self-compassionate reactions | Useful for applied scenario measurement; example development of Japanese version. [8] |
| Compassionate Engagement and Action Scales (CEAS) | Compassion for self, compassion to others, and compassion from others; each with engagement and action components | Six scales (three orientations x engagement/action); 10-point Likert; engagement 6 items; action 4 items per orientation | Compute engagement and action scores; higher = greater compassionate motivation/behaviour | Captures multiple compassion "flows" relevant to clinical work; validation available in caregiving samples. [9,10] |
| Fears of Compassion Scales (FoCS) | Barriers and fears related to compassion for self, for others, and from others | Three subscales; Likert response; items assess avoidance/fear of affiliative emotions | Higher scores indicate greater fear/avoidance of compassion | Useful when interventions may activate resistance to compassion; relevant in high self-criticism. [11] |
| Forms of Self-Criticizing/Attacking and Self-Reassuring Scale (FSCRS) | Self-criticism (inadequate self, hated self) and self-reassurance | 22 items; 0-4 Likert; 3-factor structure | Subscale means or sums; higher self-reassurance indicates more supportive self-relating; higher self-criticism indicates harsher self-relating | Commonly used with compassion-focused and ACT studies; strong normative and psychometric evidence. [12,13] |
| FSCRS Short Form (FSCRS-SF) | Brief assessment of self-criticism and self-reassurance | 14 items; retains 3-factor structure | Subscale scores analogous to full FSCRS | Suitable when brevity is needed; validated for research contexts requiring short measures. [14] |
| Mindful Self-Care Scale (MSCS) | Mindful self-care behaviours (includes self-compassionate self-care practices) | 33 items (+ optional 3 general items); multiple self-care domains | Domain and total scores; higher indicates more frequent mindful self-care practices | Useful when self-compassion is considered alongside self-care routines; validated in palliative care providers. [15-17] |
| Brief Mindful Self-Care Scale (B-MSCS) | Brief mindful self-care behaviours | Short form derived from MSCS (brief version) | Total and/or domain scores depending on version | Recommended when repeated assessments are planned and respondent burden is a concern. [16] |
| Sussex-Oxford Compassion Scales (SOCS-S; SOCS-O) | Compassion for self and compassion for others (five-dimension model) | Two 20-item scales; 5-point Likert; 5 dimensions (recognizing suffering, universality, feeling, tolerating, acting) | Total and dimension scores; higher indicates greater compassion | Strong psychometric development; useful for separating self- and other-compassion. [18] |
| Compassion Scale (CS) - Pommier | Compassion for others (Neff-based model) | 16 items; kindness, common humanity, mindfulness, and reduced indifference | Total and subscale scores; higher indicates greater compassion toward others | Suitable for studies focused on other-compassion rather than self-compassion. [19] |
| Compassion Scale (10-item) - Martins | General compassion (brief trait measure) | 10 items; self-report; brief trait assessment | Mean or sum; higher indicates greater compassion | Provides a brief alternative; psychometric evaluation available with clinical and community samples. [20] |
| Santa Clara Brief Compassion Scale (SCBCS) | Compassion toward strangers/humankind | 5 items; 7-point Likert; brief measure derived from compassionate love items | Sum or mean; higher indicates greater compassion toward others | Useful in large surveys and mindfulness research due to brevity. [21] |
| Compassionate Love Scale (CLS) | Compassionate or altruistic love toward close others or humanity | 21 items; alternative forms for target (close others vs humanity) | Mean score; higher indicates greater compassionate love | Often used as convergent measure in compassion psychometrics. [22] |
| Schwartz Center Compassionate Care Scale (SCCCS) | Patient-rated compassionate healthcare behaviours | 12 items; 10-point response options in original version | Total score; higher indicates greater perceived clinician compassion | Useful for service evaluation and clinician feedback; psychometric and Rasch evidence available. [23,24] |
| Professional Quality of Life Scale (ProQOL-5) | Compassion satisfaction, burnout, and secondary traumatic stress (helping professions) | 30 items; 5-point Likert; 3 subscales | Subscale scores; higher compassion satisfaction indicates positive professional quality; higher burnout/STS indicates greater fatigue | Widely used for compassion-related occupational outcomes; revised short forms exist. [25] |
| ProQOL-21 (revised short form) | Shortened professional quality of life profile (CS, burnout, STS) | 21 items; psychometric refinement of ProQOL | Subscale scores analogous to ProQOL | Useful when shorter occupational assessment is needed; validation in large samples. [26] |
| Compassion Competence Scale (nursing) | Compassion competence in nurses (communication, sensitivity, insight) | 17 items; 5-point Likert; 3 domains | Total and domain scores; higher indicates greater compassion competence | Clinically oriented measure for healthcare professionals; development and validation available. [27] |

*Legend: SCS = Self-Compassion Scale; SCS-SF = Self-Compassion Scale-Short Form; SSCS = State Self-Compassion Scale; BSCI = Brief Self-Compassion Inventory; SCS-Y = Self-Compassion Scale for Youth; SCCS = Self-Compassion and Self-Criticism Scales; SCRI = Self-Compassionate Reactions Inventory; CEAS = Compassionate Engagement and Action Scales; FoCS = Fears of Compassion Scales; FSCRS = Forms of Self-Criticizing/Attacking and Self-Reassuring Scale; MSCS = Mindful Self-Care Scale; SOCS = Sussex-Oxford Compassion Scales; CS = Compassion Scale; SCBCS = Santa Clara Brief Compassion Scale; CLS = Compassionate Love Scale; SCCCS = Schwartz Center Compassionate Care Scale; ProQOL = Professional Quality of Life.*
